# Evaluation of the Therapeutic and Economic Impact of Adalimumab Versus Methotrexate in the Management of Psoriasis: A Prospective Comparative Study

**DOI:** 10.7759/cureus.98411

**Published:** 2025-12-03

**Authors:** Kajomi Shingala, Dipesh Nariya, Nilesh Chavda, Purvik Busa, Deval Vora

**Affiliations:** 1 Department of Dermatology, Shri M. P. Shah Government Medical College, Jamnagar, IND; 2 Department of Pharmacology, Shri M. P. Shah Government Medical College, Jamnagar, IND

**Keywords:** adalimumab, biologics, cost-effectiveness, methotrexate, pharmacoeconomics, psoriasis

## Abstract

Background: Psoriasis is a chronic inflammatory skin disease often requiring systemic therapy for moderate-to-severe cases. Methotrexate (MTX) is a cost-effective conventional treatment, while adalimumab (ADL), a tumor necrosis factor-alpha (TNF-α) inhibitor, offers higher efficacy but at a significantly higher cost. Comparative real-world data on their effectiveness, safety, and cost-efficiency in resource-limited settings remain scarce.

Aim: In the present study, we aimed to compare the therapeutic efficacy, safety profile, and cost-effectiveness of ADL versus MTX in the treatment of moderate-to-severe plaque psoriasis.

Methods: This prospective, observational study was conducted with 80 (100%) adult patients, divided equally into the ADL group (n=40, 50%) and the MTX group (n=40, 50%). Patients were evaluated over 24 weeks for clinical efficacy (Psoriasis Area Severity Index (PASI) 75/90 response rates), safety (adverse drug reactions (ADRs)), quality of life (Dermatology Life Quality Index (DLQI) scores), and pharmacoeconomic parameters, including average cost-effectiveness ratio (ACER) and incremental cost-effectiveness ratio (ICER).

Results: At week 24, PASI 75 and PASI 90 responses were significantly higher in the ADL group (n=25, 62.16% and n=11, 27% respectively) compared to the MTX group (n=10, 23.7% and n=1, 2.63% respectively) (p<0.05). ADL showed greater mean PASI reduction (8.95 vs. 6.58; p<0.001) and DLQI improvement (8.22 vs. 4.89; p<0.001). Adverse events were similar between groups. However, ADL incurred a markedly higher treatment cost (₹97,500 vs. ₹396.4). The ACER per PASI 75 responder was ₹156,784.42 for ADL versus ₹1,672.15 for MTX. ICER for an additional PASI 75 responder with ADL was ₹252,553.97.

Conclusions: ADL demonstrated superior clinical efficacy and quality-of-life improvement over MTX but at a substantially higher cost. Despite its effectiveness, ADL’s economic burden limits its feasibility in resource-constrained settings. MTX remains the more cost-effective option, underscoring the need for affordability-enhancing measures for biologics in India.

## Introduction

Psoriasis is a chronic, immune-mediated inflammatory skin disease affecting approximately 2-3% of the global population, with significant implications for physical and mental well-being [1,2]. It is characterized by hyperproliferation of keratinocytes and immune dysregulation, driven primarily by T-cell activation and pro-inflammatory cytokines such as tumor necrosis factor-alpha (TNF-α), interleukin (IL)-17, and IL-23 [3]. Psoriasis manifests in various clinical forms, with plaque psoriasis (psoriasis vulgaris) being the most common, affecting nearly 80-90% of patients [4]. The disease often presents with erythematous, scaly plaques, commonly on the scalp, elbows, knees, and lower back, significantly impairing quality of life [4]. 

Beyond cutaneous symptoms, psoriasis is increasingly recognized as a systemic inflammatory disorder associated with multiple comorbidities, including psoriatic arthritis (PsA), cardiovascular disease, metabolic syndrome, obesity, diabetes mellitus, and depression [5,6]. The chronic inflammatory burden of psoriasis underscores the need for effective, long-term management strategies that balance efficacy and safety in systemic therapies. Systemic treatment is recommended for moderate-to-severe psoriasis (i.e., affecting >10% of body surface area, Psoriasis Area Severity Index (PASI) score >10, or significant impact on quality of life) when topical therapies and phototherapy fail. Given the chronic nature of psoriasis and the cost disparity between biologics and traditional systemic agents, evaluating cost-effectiveness in routine Indian practice is crucial for sustainable care delivery [7].

Methotrexate (MTX), a folic acid antagonist, has been used in psoriasis management since the 1950s. It inhibits dihydrofolate reductase, thereby reducing DNA synthesis and suppressing T-cell activity, leading to reduced keratinocyte proliferation and inflammation [8]. Despite its long history and cost-effectiveness, MTX requires close monitoring due to its dose-dependent toxicities, including hepatotoxicity, myelosuppression, gastrointestinal symptoms, and pulmonary fibrosis [9]. Additionally, its slow onset of action and variable response rates (with PASI 75 responses of ~35-50% at 12-16 weeks) often limit its use as a first-line option for many patients [10]. 

Adalimumab (ADL) is a fully human monoclonal antibody targeting TNF-α, a key pro-inflammatory cytokine in psoriasis pathogenesis. It binds to TNF-α, preventing its interaction with TNF receptors, thereby reducing inflammation and keratinocyte proliferation [11]. Clinical trials, such as the REVEAL (Randomized Controlled Evaluation of Adalimumab Every-Other-Week Dosing in Moderate to Severe Psoriasis Trial) and CHAMPION (Comparative study of Humira vs. Methotrexate vs. Placebo in Psoriasis Patients) studies, have demonstrated that ADL achieves PASI 75 responses in up to 71% of patients within 16 weeks, making it significantly more effective than conventional systemic therapies, including MTX [12,13]. Additionally, ADL is associated with a faster onset of action, higher sustained remission rates, and lower cumulative toxicity compared to MTX [14]. However, it carries the risk of infections, immunogenicity, injection site reactions, and potential cardiovascular risks [15]. 

Although both ADL and MTX are widely used in psoriasis treatment, head-to-head comparative studies evaluating their efficacy and safety remain limited. While ADL offers higher efficacy and better tolerability, its higher cost and potential long-term immunogenicity limit its widespread adoption in resource-constrained settings [7]. On the other hand, MTX remains a cost-effective option, but its long-term toxicity concerns necessitate careful patient selection and monitoring. 

Pharmacoeconomic analyses help healthcare providers, policymakers, and insurance companies determine the value of therapies by comparing their clinical effectiveness with their economic impact. This is particularly important in conditions like psoriasis, where high-cost biologics may offer superior efficacy but pose financial challenges, necessitating a balance between treatment benefits and affordability [13].

Given these differences, this study aimed to directly compare the safety and efficacy of ADL versus MTX in the treatment of psoriasis. The objectives were: (i) to assess clinical efficacy based on PASI 75 and PASI 90 response rates; (ii) to evaluate the safety profile of both drugs, focusing on hepatotoxicity, infections, and immunogenicity; (iii) to analyze patient-reported outcomes, including Dermatology Life Quality Index (DLQI) scores and treatment adherence; and (iv) to perform pharmacoeconomic analysis comparing cost-effectiveness of the two treatments. By addressing these aspects, this study sought to provide valuable clinical insights to guide dermatologists in optimizing systemic therapy selection for psoriasis patients. Specifically, it aimed to compare the efficacy, safety, quality-of-life improvement, and cost-effectiveness of ADL and MTX in patients with moderate-to-severe plaque psoriasis over a 24-week period.

## Materials and methods

Study design and population 

This open-label, prospective, observational single-center study was conducted at Shri M. P. Shah Government Medical College, Jamnagar, India, focusing on patients with moderate-to-severe plaque psoriasis (diagnosed by clinical examination and confirmed with biopsy) who required systemic therapy. The study aimed to compare the safety, efficacy, and cost-effectiveness of ADL and MTX in routine clinical practice. Due to the availability and accessibility constraints of ADL in government hospitals, patients were not randomly assigned but were prescribed treatment based on the physician's discretion and patient eligibility. A total of 80 patients were included, with an equal distribution of patients receiving either ADL or MTX in a 1:1 ratio. The equal distribution was chosen to enhance comparability across treatments, minimize potential bias from unequal group sizes, and improve the interpretability of statistical analysis. 

Eligible patients were between the ages of 18 and 65 years, had no prior contraindications to either therapy, and provided written informed consent. Individuals with severe hepatic or renal dysfunction and a history of active tuberculosis, malignancy, or chronic infections were excluded from the study. Additionally, pregnant and lactating women and those using other systemic immunosuppressants were excluded. The study was conducted per the Declaration of Helsinki and Good Clinical Practice (GCP) guidelines, and approval was obtained from the Institutional Ethics Committee of Shri M. P. Shah Government Medical College (42/01/2025). The study was started on February 28, 2025, and completed on September 30, 2025 (seven months), including patient recruitment, treatment, and follow-up periods. 

Patients in the ADL group received an 80 mg subcutaneous injection for the initial dose, followed by 40 mg subcutaneous injections every two weeks (following the standard psoriasis protocol), while those in the MTX group were started on 7.5-15 mg orally per week, with dose adjustment based on individual response and tolerability. Patients receiving MTX were also prescribed folic acid (5 mg per week) to minimize drug-related toxicities. 

Baseline assessments, including demographic data, medical history, disease duration, prior treatments, baseline DLQI scores, and baseline PASI scores, were recorded. Follow-up visits were scheduled at weeks four, eight, 12, 16, and 24, during which clinical response, adverse drug reactions (ADRs), clinical outcomes, and cost-effectiveness data were collected. 

Outcome measures and assessment tools

The primary outcomes of this study included efficacy and safety assessments. Efficacy was measured by the proportion of patients achieving PASI 75 and PASI 90 at week 24, where PASI 75 indicates a ≥75% reduction in PASI score from baseline and PASI 90 signifies ≥90% reduction, reflecting near-complete disease clearance. Safety was assessed based on the incidence of ADRs.

Secondary outcomes included changes in DLQI scores, which assess the impact of psoriasis on daily life, and treatment adherence rates, analyzed through discontinuation due to adverse effects or lack of efficacy. Additionally, a cost-effectiveness analysis was performed by calculating the total cost per PASI 75 responder, the average cost-effectiveness ratio (ACER), and the incremental cost-effectiveness ratio (ICER) for both treatments. ACER is important for describing the overall efficiency of a single intervention, while ICER is crucial for comparing alternatives and guiding real-world healthcare decision-making.

Disease severity was evaluated using PASI, and health-related quality of life was assessed using DLQI. PASI is a clinician-rated composite score originally described by Fredriksson and Pettersson in 1978, and is publicly available for use in academic and clinical research without license restriction [16]. DLQI, developed by Finlay and Khan in 1994, is a copyright-protected instrument owned by Professor Andrew Finlay (Cardiff University) [17]. Formal permission to use the DLQI in the present study was obtained through the official Cardiff University licensing system.

Sample size calculation 

The sample size was calculated based on expected PASI 75 response rates of 71% for ADL and 40% for MTX, as reported in previous literature [13]. Using a two-sided α = 0.05 and a power (1-β) of 80%, the required sample size per group was estimated. Based on the calculation, 35 patients per group were required. To account for a 10% dropout, the final sample size was set at 40 patients per group, totaling 80 patients. 

Statistical analysis 

Descriptive statistics were used to summarize baseline characteristics. Continuous variables (e.g., age, baseline PASI, baseline DLQI, and treatment duration) were assessed for normality using the Shapiro-Wilk test. Normally distributed variables were expressed as mean ± standard deviation (SD) and compared between groups using the independent samples Student’s t-test. Variables such as baseline PASI and DLQI scores showed non-normal distribution, while age and treatment duration were normally distributed. Continuous variables that did not meet normality assumptions were summarized as median and interquartile range (IQR) and compared using the Mann-Whitney U test. Within-group changes in PASI and DLQI were analyzed using paired t-tests for normally distributed variables or Wilcoxon signed-rank tests for non-normal variables.

For continuous variables not meeting normality assumptions, the Mann-Whitney U test was applied for between-group comparisons. Categorical variables, including gender, PASI response rates, and ADR incidence, were presented as frequency (%). For between-group comparisons, the chi-square test was applied for categorical variables, while an independent t-test was used for continuous data. Within-group comparisons for changes in PASI and DLQI scores were analyzed using paired t-tests. Categorical variables, including PASI 75/90 response rates and ADR incidence, were presented as frequency (%) and analyzed using the chi-square test or Fisher’s exact test where appropriate. Statistical significance was set at p<0.05. All analyses were performed using R v4.2 (R Foundation for Statistical Computing, Vienna, Austria).

Normality of continuous variables was assessed using the Shapiro-Wilk test. Age and treatment duration followed a normal distribution (p>0.05). In contrast, baseline PASI scores, baseline DLQI scores, and changes in PASI and DLQI over time demonstrated a non-normal distribution (p<0.05). Accordingly, parametric or non-parametric tests were selected based on these results.

Disease severity was assessed using PASI, which combines assessments of erythema, induration, and desquamation across four body regions (head, trunk, upper limbs, lower limbs), each weighted by area of involvement. PASI scores range from 0 to 72, with higher scores indicating more severe disease: <10, mild psoriasis; 10-20, moderate psoriasis; and >20, severe psoriasis. Health-related quality of life was measured using DLQI. The DLQI consists of 10 items, each scored from 0 (not at all affected) to 3 (very much affected), yielding a total score from 0 to 30. Interpretation of DLQI scores is as follows: 0-1, no effect on the patient’s life; 2-5, small effect; 6-10, moderate effect; 11-20, very large effect; and 21-30, extremely large effect.

ACER was calculated as the total mean cost per patient divided by the mean effectiveness achieved in each group. ACER = (Mean total cost (₹) per patient) / (Mean change in effectiveness measure (e.g., PASI improvement or PASI 75 responders)). The ACER thus represents the cost per unit improvement in PASI or DLQI for each treatment arm individually.

For the cost-effectiveness analysis, the total cost per PASI 75 responder and the total cost per PASI score reduction were calculated, and the ICER was derived. ICER = (Cost ADL - Cost MTX) / (Effectiveness ADL - Effectiveness MTX). Pharmacoeconomic analysis was conducted keeping in mind the patient, hospital, and societal perspectives since India has a complex healthcare system involving both the government and private settings. 

'Effectiveness ADL' and 'Effectiveness MTX' denote the mean change in PASI score from baseline (ΔPASI) or, alternatively, the proportion of patients achieving PASI 75 (≥75% improvement from baseline). The ICER was expressed as cost per additional responder achieving PASI 75 with ALD compared to MTX. A sensitivity analysis was conducted to assess variations in cost estimates (considering ±10% cost variation). All statistical analyses were performed using R v4.2, with p<0.05 considered statistically significant. 

## Results

**Figure 1:**
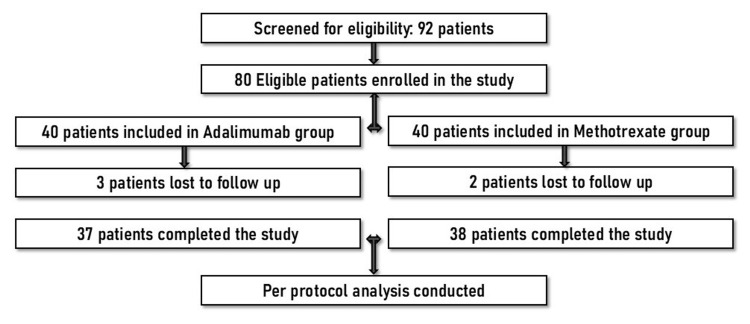
Participant recruitment and analysis

A total of 80 patients were enrolled in the study, with 40 patients assigned to the ADL group and 40 patients to the MTX group. A total of five patients were lost to follow-up (Figure 1). The sex distribution was comparable between the two groups. The mean age at baseline was 38.8 ± 8.4 years in the ADL group and 37.5 ± 7.6 years in the MTX group. The mean disease duration was comparable between the MTX group (6.1 ± 3.7 years) and the ADL group (5.1 ± 2.7 years). PsA diagnosis was noted in 13.5% (n=5) of ADL-treated patients and 18.4% (n=7) of MTX-treated patients. The mean BMI was comparable between the groups, at 27.5 ± 4.7 kg/m² in the ADL group and 28.8 ± 3.0 kg/m² in the MTX group.

The mean baseline PASI score was slightly lower in the ADL group (11.41 ± 3.41) compared to the MTX group (12.06 ± 3.89), with no significant difference (p=0.443, independent t-test). The mean baseline DLQI score was 9.83 ± 3.25 in the ADL group and 10.32 ± 2.64 in the MTX group, with no significant difference (p=0.475, independent t-test) (Table 1).

**Table 1:** Baseline characteristics Independent t-tests for continuous variables (age, duration, BMI, PASI, DLQI) and chi-square tests (χ²) for categorical variables (sex, PsA, smoking, alcohol) were applied. BMI, body mass index; PASI, Psoriasis Area and Severity Index; DLQI, Dermatology Life Quality Index; PsA, psoriatic arthritis; SD, standard deviation

Parameter		Adalimumab group (N = 37)	Methotrexate group (N = 38)	P-value	Test statistic
Sex	Male (n)	27	27	0.79	χ² = 0.07
	Female (n)	10	11		
Age at baseline in years (mean ± SD)		38.8 ± 8.4	37.5 ± 7.6	0.53	t = 0.63
Duration of disease in years (mean ± SD)		5.1 ± 2.7	6.1 ± 3.7	0.19	t = -1.33
Diagnosed with PsA (n (%))		5 (13.5)	7 (18.4)	0.56	χ² = 0.35
BMI in kg/m² (mean ± SD)		27.5 ± 4.7	28.8 ± 3.0	0.17	t = -1.39
Smokers (n (%))		7 (18.9)	8 (21)	0.81	χ² = 0.06
Alcohol use (n (%))		5 (13.5)	3 (7.9)	0.46	χ² = 0.55
PASI score (mean ± SD)		11.41 ± 3.41	12.06 ± 3.89	0.44	t = -0.77
DLQI score (mean ± SD)		9.83 ± 3.25	10.32 ± 2.64	0.48	t = -0.71

**Figure 2:**
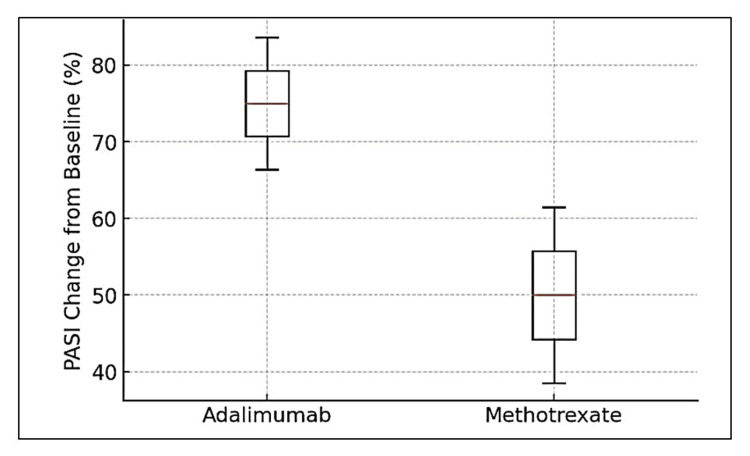
PASI score comparison The box plot illustrates the percentage improvement in PASI scores from baseline at week 24 for patients treated with adalimumab and methotrexate. The median PASI reduction was significantly higher in the adalimumab group (75%) compared to the methotrexate group (50%) (p<0.001, independent t-test). The IQR for adalimumab was 66.4%-83.6%, while for methotrexate, it was 38.5%-61.5%. The figure highlights the superior efficacy of adalimumab in achieving PASI improvement compared to methotrexate. PASI: Psoriasis Area and Severity Index; IQR, interquartile range

At week 24, a PASI 75 response was achieved in 62.16% (n=23) of patients in the ADL group compared to 23.7% (n=9) in the MTX group. Similarly, the proportion of patients achieving PASI 90 at week 24 was significantly higher in the ADL group (27%; n=10) than in the MTX group (2.63%; n=1) (p=0.007, chi-square test) (Figure 2).

The median percentage change in PASI from baseline was significantly greater in the ADL group (75% (IQR 66.4, 83.6)) compared to the MTX group (50% (IQR 38.5, 61.5)). The mean absolute PASI reduction from baseline was also significantly higher in the ADL group (8.95 ± 1.45) compared to the MTX group (6.58 ± 2.05) (p<0.001, independent t-test). 

The improvement in quality of life, as measured by the DLQI change from baseline, was significantly greater in the ADL group, with a mean reduction of 8.22 ± 2.61, compared to 4.89 ± 1.89 in the MTX group (p<0.001, independent t-test).

A total of 14 adverse events were reported in the ADL group, whereas 16 adverse events were recorded in the MTX group, with no statistically significant difference (p=0.715, chi-square test). Injection site reaction, pain, and fever were common adverse events associated with ADL, whereas gastrointestinal (GI) upset and oral mucosal ulcer were commonly associated with MTX. No serious adverse events leading to discontinuation were noted in either group (Table 2).

**Table 2:** Therapeutic assessment Continuous data (mean ± SD) were analyzed using independent t-tests (t), median (IQR) data using the Mann-Whitney U test (U), and categorical data (n (%)) using chi-square tests (χ²) as appropriate. Test statistics are reported in the table. PASI, Psoriasis Area and Severity Index; DLQI, Dermatology Life Quality Index; IQR, interquartile range; SD, standard deviation

Efficacy outcomes		Adalimumab group (N = 37)	Methotrexate group (N = 38)	P-value	Test statistic
Primary outcomes	PASI change from baseline (%) (median (IQR))	75 (66.4, 83.6)	50 (38.5, 61.5)	<0.001	U = 94.5
	PASI reduction (mean ± SD)	8.95 ± 1.45	6.58 ± 2.05	<0.001	t = 5.35
	PASI 75 at week 24 (n (%))	23 (62.16)	9 (23.7)	0.013	χ² = 6.20
	PASI 90 at week 24 (n (%))	10 (27)	1 (2.63)	0.007	χ² = 7.20
Secondary outcomes	DLQI change from baseline (mean ± SD)	8.22 ± 2.61	4.89 ± 1.89	<0.001	t = 5.04
Safety outcomes	Total number of adverse events	14	16	0.715	χ² = 0.13

Cost calculation

The average cost of ADL (40 mg per prefilled syringe) was determined based on pricing from three different sources as per the prices on February 28, 2025: MrMed, Healthkind Pharma, and IndiaMART [18-20]. The individual prices from these sources were averaged to obtain a mean price of ₹7,500 per syringe. For the 24-week treatment period, each patient required a total dose of 520 mg, equivalent to 13 syringes. Multiplying the average price per syringe by the required number of syringes resulted in a total treatment cost of ₹97,500 per patient. 

For MTX, pricing was sourced from Pharmeasy, Netmeds, and Chemist Online as per the prices on February 28, 2025 [21-23]. The cost per 10-tablet pack varied across these sources, and an average price was calculated to be ₹100.1 per pack. Given that each patient required a total dose of 297 mg over 24 weeks, the corresponding number of tablets was rounded up to 40. The total cost for the required dosage was then derived based on the average price per 10-tablet pack, resulting in a treatment cost of ₹396.4 per patient (Table 3). Costs were analyzed from the patient's out-of-pocket perspective in 2025 Indian rupees. 

**Table 3:** Cost of available formulations in India INR, Indian rupees

Therapy	Source	Price INR	Quantity	Price per unit
Adalimumab (40 mg)	MrMed [18]	₹7,700.00	1 prefilled syringe	₹7,700.00
	Healthkind Pharma [19]	₹7,800.00	1 prefilled syringe	₹7,800.00
	IndiaMART [20]	₹7,000.00	1 prefilled syringe	₹7,000.00
	Average price	₹7,500.00	-	₹7,500.00
Methotrexate (7.5 mg)	Pharmeasy [21]	₹97.00	10 tablets	₹9.70
	Netmeds [22]	₹163.25	10 tablets	₹16.33
	Chemist Online [23]	₹40.00	10 tablets	₹4.00
	Average price	₹100.1	-	₹10.01

Treatment costs

The total cost of ADL (40 mg) per patient over 24 weeks was ₹97,500, based on an average price of ₹7,500 per prefilled syringe and a total dosage requirement of 520 mg (13 units). For MTX (7.5 mg), the total cost per patient over 24 weeks was ₹396.4, based on an average price of ₹100.1 per 10 tablets and a total dosage requirement of 297 mg (39.6 units) (Table 4).

**Table 4:** Total cost of therapy *Total cost of treatment = (Average price) × (Total no. of units required)

Therapy	Total average dose per patient (24 weeks)	Total cost of treatment*
Adalimumab	520 mg (13 units)	₹97,500
Methotrexate	297 mg (39.6 units)	₹396.4

Cost-effectiveness analysis

The ACER for achieving PASI 75 was significantly higher for ADL (₹156,784.42 per PASI 75 responder) compared to MTX (₹1,672.15 per PASI 75 responder). The ICER for achieving an additional PASI 75 responder with ADL compared to MTX was ₹252,553.97.

The ACER per PASI score reduction was ₹10,892.74 for ADL, while it was ₹60.25 for MTX. The ICER for an additional unit of PASI score reduction with ADL over MTX was ₹41,004.06. 

While ADL demonstrated superior efficacy in achieving PASI 75 and PASI score reduction, its cost per responder was significantly higher compared to MTX. The ICER values suggest that the additional clinical benefit of ADL comes at a substantial financial burden (Table 5). Sensitivity analysis, varying the drug cost by ±10%, did not alter the ICER ranking between groups.

**Table 5:** Cost-effectiveness analysis 'Additional PASI 75 responders' refers to the incremental number of patients achieving a ≥75% reduction in PASI score with adalimumab compared to methotrexate. 'Additional PASI score reduction' denotes the incremental improvement in mean PASI score achieved with adalimumab over methotrexate. ACER, average cost-effectiveness ratio; ICER, incremental cost-effectiveness ratio; PASI, Psoriasis Area Severity Index

Cost-effectiveness analysis		Adalimumab	Methotrexate
Based on the PASI 75 responder rate	ACER	₹156,784.42/PASI 75 responder	₹1,672.15/PASI 75 responder
	ICER	₹252,553.97/additional PASI 75 responders	
Based on the mean PASI score reduction	ACER	₹10,892.74/PASI score reduction	₹60.25/PASI score reduction
	ICER	₹41,004.06/additional PASI score reduction	

## Discussion

Psoriasis is a chronic inflammatory skin condition with a prevalence in India ranging from 0.44% to 2.8%, affecting approximately 3.59 million individuals [24]. The disease predominantly manifests in the third or fourth decade of life and is twice as common in male patients compared to female patients. This significant prevalence underscores the necessity for effective and accessible treatment options tailored to the Indian demographic [24].

Our study demonstrated that ADL significantly outperformed MTX in treating moderate-to-severe plaque psoriasis. At week 24, 62.16% of patients in the ADL group achieved a PASI 75 response, compared to 23.7% in the MTX group (p=0.013). Additionally, 27% of ADL-treated patients reached PASI 90, whereas only 2.63% of MTX-treated patients did so (p=0.007). These findings align with previous studies, such as the one by Armstrong et al., which reported that patients on ADL were twice as likely to be clear or nearly clear of psoriasis compared to those on MTX [25]. Similarly, previous studies have found that ADL treatment is associated with improved PASI 75 outcomes compared to MTX [13,26].

The improvement in quality of life, as measured by the DLQI, was more substantial in the ADL group, with a mean reduction of 8.22 ± 2.61, compared to 4.89 ± 1.89 in the MTX group (p<0.001). These results are consistent with previous findings, where ADL led to significantly greater improvements in DLQI scores compared to MTX or placebo [27].

Both treatments exhibited comparable safety profiles. The ADL group reported 14 adverse events, while the MTX group reported 16, with no statistically significant difference (p=0.715). No serious adverse events leading to discontinuation were noted in either group. This aligns with existing literature, which indicates that both ADL and MTX have acceptable and comparable safety profiles [13,28].

Pharmacoeconomic studies guide evidence-based decision-making, helping optimize healthcare budgets, improve patient access to essential medications, and enhance overall public health outcomes. Two key metrics used in cost-effectiveness analysis are the ICER and the ACER. ICER quantifies the additional cost required to achieve one extra unit of health benefit (e.g., cost per additional PASI 75 responder). It is particularly useful when comparing a new, more expensive treatment to a standard, cost-effective alternative [29].

The total cost of ADL per patient over 24 weeks was ₹97,500, while MTX cost ₹396.4. The ACER for achieving PASI 75 was ₹156,784.42 per responder for ADL and ₹1,672.15 for MTX. The ICER for achieving an additional PASI 75 responder with ADL compared to MTX was ₹252,553.97. A study analyzing the cost per responder of biologics in moderate-to-severe plaque psoriasis in India reported that the annual cost of ADL was ₹188,730 [30]. While ADL offers superior efficacy, its high cost poses a challenge for widespread adoption in India. This necessitates a careful evaluation of budgetary impact and consideration of cost-effective alternatives [30].

Our study highlights the superior efficacy of ADL over MTX in achieving PASI 75 and PASI 90 responses in patients with moderate-to-severe plaque psoriasis. Additionally, ADL demonstrated a greater improvement in quality of life (DLQI scores), reinforcing its role as a more effective treatment option. While both treatments had comparable safety profiles, the economic burden of ADL, as reflected in the ICER and ACER analyses, raises concerns regarding its accessibility and feasibility for widespread use. Given the significant cost disparity, MTX remains the more cost-effective option under both PASI 75 responder analysis and absolute PASI reduction analysis, reducing disease burden per unit cost. Sensitivity analysis showed stable ICER estimates, reinforcing the reliability of the cost-effectiveness findings.

The limitations of our study include a relatively small sample size and a short follow-up period of 24 weeks. Long-term studies with larger cohorts are necessary. Allocation bias could be avoided using appropriate methods for matching or stratification. The dose variations need to be thoroughly analyzed for better representation of the data. Future studies should consider the cost of visits, folic acid supplementation, and laboratory monitoring. Additionally, future research should focus on strategies to reduce the cost of biologics, such as introducing biosimilars, to enhance their accessibility in resource-limited settings. Future pharmacoeconomic studies incorporating quality-adjusted life years (QALY) estimation and societal cost perspectives could further inform evidence-based psoriasis management in resource-limited settings.

## Conclusions

The findings from our study underscore the urgent need for cost-reduction strategies, including the adoption of biosimilars and insurance support, to improve access to biologics in India. While ADL provides clear clinical benefits, its budgetary implications necessitate careful consideration by healthcare policymakers when formulating psoriasis treatment guidelines. Ultimately, a balanced approach incorporating both clinical efficacy and economic feasibility is essential to optimize treatment strategies for psoriasis, ensuring that patients receive effective and affordable care in diverse healthcare settings.

## References

[1] Boehncke WH, Schön MP (2015). Psoriasis. Lancet.

[2] Rachakonda TD, Schupp CW, Armstrong AW (2014). Psoriasis prevalence among adults in the United States. J Am Acad Dermatol.

[3] Lowes MA, Bowcock AM, Krueger JG (2007). Pathogenesis and therapy of psoriasis. Nature.

[4] Griffiths CE, Barker JN (2007). Pathogenesis and clinical features of psoriasis. Lancet.

[5] Mehta NN, Yu Y, Pinnelas R, Krishnamoorthy P, Shin DB, Troxel AB, Gelfand JM (2011). Attributable risk estimate of severe psoriasis on major cardiovascular events. Am J Med.

[6] Nisa N, Qazi MA (2010). Prevalence of metabolic syndrome in patients with psoriasis. Indian J Dermatol Venereol Leprol.

[7] Armstrong AW, Puig L, Joshi A (2020). Comparison of biologics and oral treatments for plaque psoriasis: a meta-analysis. JAMA Dermatol.

[8] Warren RB, Weatherhead SC, Smith CH, Exton LS, Mohd Mustapa MF, Kirby B, Yesudian PD (2016). British Association of Dermatologists' guidelines for the safe and effective prescribing of methotrexate for skin disease 2016. Br J Dermatol.

[9] Pathirana D, Ormerod AD, Saiag P (2009). European S3-guidelines on the systemic treatment of psoriasis vulgaris. J Eur Acad Dermatol Venereol.

[10] Flytström I, Stenberg B, Svensson A, Bergbrant IM (2008). Methotrexate vs. ciclosporin in psoriasis: effectiveness, quality of life and safety. A randomized controlled trial. Br J Dermatol.

[11] Lynch M, Kirby B, Warren RB (2014). Treating moderate to severe psoriasis - best use of biologics. Expert Rev Clin Immunol.

[12] Menter A, Tyring SK, Gordon K (2008). Adalimumab therapy for moderate to severe psoriasis: a randomized, controlled phase III trial. J Am Acad Dermatol.

[13] Saurat JH, Stingl G, Dubertret L (2008). Efficacy and safety results from the randomized controlled comparative study of adalimumab vs. methotrexate vs. placebo in patients with psoriasis (CHAMPION). Br J Dermatol.

[14] Menter A, Strober BE, Kaplan DH (2019). Joint AAD-NPF guidelines of care for the management and treatment of psoriasis with biologics. J Am Acad Dermatol.

[15] Takeshita J, Grewal S, Langan SM, Mehta NN, Ogdie A, Van Voorhees AS, Gelfand JM (2017). Psoriasis and comorbid diseases: epidemiology. J Am Acad Dermatol.

[16] Fredriksson T, Pettersson U (1978). Severe psoriasis - oral therapy with a new retinoid. Dermatologica.

[17] Finlay AY, Khan GK (1994). Dermatology Life Quality Index (DLQI) - a simple practical measure for routine clinical use. Clin Exp Dermatol.

[18] (2025). MrMed. Adalipca 40 mg injection. https://www.mrmed.in/medicines/adalipca-40mg-injection.

[19] (2025). Healthkind Pharma. Adalirel 40 mg adalimumab 40 mg injection. https://www.healthkindpharma.com/adalimumab.html.

[20] (2025). IndiaMART. Adalimumab 40 mg injection. https://www.indiamart.com/proddetail/adalimumab-40mg-injection-24206701312.html.

[21] (2025). PharmEasy. Mtx 7.5 mg strip of 10 tablets. https://pharmeasy.in/online-medicine-order/mtx-7-5mg-tablet-10-s-226049.

[22] (2025). Netmeds. Folitrax 7.5 mg tablet 10's. https://www.netmeds.com/prescriptions/folitrax-7-5mg-tablet-10-s.

[23] (2025). Chemist Online. Methotrexate tablets IP 7.5 mg. https://chemistonline.in/shop/janaushadhi/methotrexate-tablets-ip-7-5-mg/.

[24] Dogra S, Yadav S (2010). Psoriasis in India: prevalence and pattern. Indian J Dermatol Venereol Leprol.

[25] Armstrong AW, Robertson AD, Wu J, Schupp C, Lebwohl MG (2013). Undertreatment, treatment trends, and treatment dissatisfaction among patients with psoriasis and psoriatic arthritis in the United States: findings from the National Psoriasis Foundation surveys, 2003-2011. JAMA Dermatol.

[26] Maurelli M, Girolomoni G, Gisondi P (2023). Cost per responder of adalimumab biosimilars versus methotrexate in patients with psoriasis: a real-life experience. J Dermatolog Treat.

[27] Prussick R, Unnebrink K, Valdecantos WC (2015). Efficacy of adalimumab compared with methotrexate or placebo stratified by baseline BMI in a randomized placebo-controlled trial in patients with psoriasis. J Drugs Dermatol.

[28] West J, Ogston S, Foerster J (2016). Safety and efficacy of methotrexate in psoriasis: a meta-analysis of published trials. PLoS One.

[29] Sanders GD, Neumann PJ, Basu A (2016). Recommendations for conduct, methodological practices, and reporting of cost-effectiveness analyses: second panel on cost-effectiveness in health and medicine. JAMA.

[30] Gokhale S, Chatterjee M, Arora S, Kawatra P (2021). Cost per responder analysis of biologics in moderate-to-severe plaque psoriasis in Indian healthcare setting. J Health Policy Outcomes Res.

